# Progression of Renal Dysfunction in Patients with Cardiovascular Disease

**DOI:** 10.2174/157340308785160543

**Published:** 2008-08

**Authors:** Yasunobu Hirata, Arihiro Kiyosue, Masao Takahashi, Hiroshi Satonaka, Daisuke Nagata, Masataka Sata, Etsu Suzuki, Ryozo Nagai

**Affiliations:** Department of Cardiovascular Medicine, University of Tokyo, 7-3-1 Hongo, Bunkyo-ku, Tokyo 113-8655, Japan

**Keywords:** CKD, angiotensin II, aldosterone, ARB, hypertension.

## Abstract

It has been established that patients with chronic kidney disease (CKD) suffer from frequent cardiovascular events. On the other hand, recent studies suggest that renal damage tends to worsen in patients with cardiovascular diseases (CVD). Although the mechanisms for the cardiorenal association are unclear, the presence of arteriosclerotic risk factors common to both CVD and CKD is important. In arteriosclerosis, vascular derangement progresses not only in the heart but also in the kidney. In addition, heart failure, cardiac catheterization and hesitation of medical treatments due to renal dysfunction may explain the progression of renal damage. Therefore, the goal of treatments is a total control of arteriosclerotic risk factors. Medication should be selected among agents with protective effects on both heart and kidney. It is important to always consider the presence of CKD for the treatment of the cardiovascular disease and strictly control the risk factors.

## INTRODUCTION

Subgroup analyses of epidemiological surveys and large-scale clinical studies have shown one after another that the presence of chronic kidney disease (CKD) worsens the prognosis of cardiovascular disease (CVD) [1, 2]. Various pathophysiological mechanisms have already been proposed as shown in Fig. (**1**). However, it is not clear whether there is a difference in the aggravation of renal function between patients with CVD and those without CVD, because there have hardly been large-scale clinical trials to prove it. Nevertheless, because such evidence has begun to accumulate, in the present review article we investigate whether renal failure tends to progress when CVD is present, and if so, discuss the pathophysiological mechanisms and the most appropriate treatment strategy.

## 1. RENAL FAILURE IN EPIDEMIOLOGICAL SURVEYS INVOLVING PATIENTS WITH CVD

The KEEP study [3] examined independent components of CKD as a cardiovascular risk state in patients presenting both disorders. Because the patients with CKD tended to die of CVD, not of renal insufficiency, this study followed 37,153 residents of 53 years old on average for a mean period of 16 months and examined the relation between CKD and CVD. During the follow-up period 7.8% of the patients suffered a myocardial infarction or cerebrovascular accident. Significant risk factors of CVD were gender male (odds ratio; 1.64), smoking (1.73), diabetes mellitus (1.66), hypertension (1.77), an estimated glomerular filtration rate (GFR) of 30-59 mL/min/1.73m^2^ (1.37), hemoglobin of less than 12.8 g/dL (1.45) and microalbuminuria of more than 30 mg/L (1.28). In comparison with the control group, the mortality increased from 3.02 in patients with only CVD and 1.98 in those with only CKD to 3.80 in those with both diseases.

Thus, it is clear that the presence of both disorders requires careful management.

In the multi institutional observation study by Levin *et al*. [4] 313 patients with CKD with an average creatinine clearance of 36 mL/min were followed for 23 months. In this study population 27% of the patients had hypertension, 18% had chronic glomerulonephritis, 25% had diabetic nephropathy and 8% had polycystic kidney disease. Moreover, 46% of the subjects had CVDs such as myocardial infarction, angina pectoris, coronary angioplasty, transient ischemic attack, cerebrovascular disorder, peripheral vascular disorder or congestive heart failure. The frequency of dialysis was significantly higher by 1.58 times in patients who concomitantly had CVD.

When both CKD and CVD were present, the two trials mentioned above showed that the prognosis was poor, but it was not absolutely clear whether CVD was a risk factor for the worsening of renal disease. O'Hare *et al*. [5] measured the ankle brachial blood pressure index (ABI) and serum creatinine concentration in 13,655 subjects of the Atherosclerosis Risk in Communities (ARIC) study. They measured serum creatinine level again three years later and examined the relation with the baseline ABI in those whose serum creatinine had increased by more than 50%. The frequency of increased creatinine was significantly greater in patients with an ABI < 0.9 by 2.16% (odds ratio 2.5) than in patients with an ABI 0.9-0.99 (0.9%) and those with an ABI >1 (0.48%). These results did not differ after the exclusion of patients with either renal insufficiency, diabetes mellitus or hypertension. The authors concluded that the presence of atherosclerosis was a risk factor for exacerbation of the renal disease.

Elsayed *et al*. [6] monitored renal function for 9.3 years on average in subjects from the ARIC study and the Cardiovascular Health Study (CHS). The patients with an increased serum creatinine concentration (by >0.4 mg/dL to 1.2 mg/dL or higher in women, or to 1.4 mg/dL in men) or a decrease of the estimated GFR (by > 15 mL/min/1.73m^2^ or to < 60 mL/min/1.73m^2^) were considered to show renal failure progression. The baseline creatinine concentration of 13,826 subjects was 0.9+/-0.2 mg/dL and the estimated GFR was 89.8+/-20.1 (SD) mL/min/1.73m^2^. Renal failure progressed in 3.8% of the subjects according to their creatinine concentration and in 2.3% according to the estimated GFR at the end of the follow up. At baseline the patients with CVD accounted for 12.9% of the study population. In patients with CVD, the odds ratio for progression of renal failure was significantly high at 1.70. The result was similar even when patients with a high level of creatinine at baseline were excluded. Risk factors such as hypertension and diabetes mellitus were present more frequently and their mobility periods were longer in patients with CVD. Thus, it was plausible that renal failure tended to progress independent of the presence of CKD when CVD was present.

## 2. MECHANISMS OF RENAL DAMAGE IN PATIENTS WITH CVD

The potential pathophysiological mechanisms for renal damage in CVD are:

### a) Presence of Risk Factors Common to CVD and CKD

Patients have many risk factors for the progression of CVD that also aggravate renal insufficiency. In the Framingham Heart study by Fox *et al*. [7] 2,585 subjects 43 years old on average who did not show renal disease were followed up for 18.5 years and the factors that influenced the new onset of renal disease were analyzed. Patients with CKD accounted for 9.4% of the population when CKD was defined as a GFR of < 59.25 mL/min/1.73m^2^ in women and < 64.25 mL/min/1.73m^2^ in men determined by the equation of MDRD. Age, GFR, BMI, diabetes mellitus, smoking, hypertension and low level of HDL-cholesterol contributed to the onset. This suggests that risk factors of CVD closely contribute to the new onset of CKD. Furthermore, the frequency of CKD increases as the number of risk factors increases. During the ARIC study CKD occurred in 7% of patients with metabolic syndrome but without diabetes mellitus, and the frequency increased significantly more in patients with metabolic syndrome than in those without it. The odds ratio was higher in subjects with more risk factors [8].

Smoking, which is an arteriosclerotic risk factor, is also a risk for renal failure. Bleyer *et al*. [9] repeated measurements of creatinine concentration more than 3 years apart in 4,142 patients 65 years of age or older. Of them, 2.8% showed elevation of the creatinine level by more than 0.3 mg/dL. This correlated with the number of cigarettes smoked, systolic blood pressure and intimal thickening of the internal carotid artery. In the MRFIT study smoking was shown to be a risk factor of end-stage renal disease [10]. The results suggested that the presence of arteriosclerotic risk factors leads to CVD and CKD.

### b) Atherosclerotic Development of the Renal Vasculature

Tracy *et al*. [11] reported that the hyalinization of kidney arterioles parallels coronary atherosclerotic progress in 25 to 54-year-old autopsy cases. Kasiske *et al*. [12] reported similar autopsy findings. They compared 57 cases with mild systemic arterial sclerosis with 57 cases that had moderate or severe sclerosis, and they found that the degree of glomerulosclerosis, arterial wall area in the kidney and glomerular size were larger in the latter group. Furthermore, the glomerular area positively correlated with heart weight and the degree of coronary atherosclerosis.

The mechanisms involved in renal failure caused by hypertension and diabetes mellitus, but not by hypercholesterolemia, have already been reviewed in detail. The deposition of lipoprotein on glomerular mesangial cells and the matrix may be important in disordered lipid metabolism [13]. In particular, oxidative LDL-cholesterol activates various growth factors and causes proliferation of mesangial cells and expansion of the matrix. Furthermore, LDL-cholesterol promotes the expression of MCP-1 chemokine and inflammation mediators such as IL-6 and NF-κB. Development of focal glomerulosclerosis and glomerular hypertrophy is associated with obesity. However, clinical diagnosis of such microvascular renal damage associated with arterial sclerosis is difficult. This disorder may account for a substantial part of the etiology-unknown end-stage renal failure in elderly persons.

## DECLINE OF RENAL PERFUSION IN THE PRESENCE OF HEART FAILURE AND ACTIVATION OF NEUROHUMORAL FACTORS

Fig. (**2**) shows the treatment course of a 41-year-old man with dilated cardiomyopathy and altered renal function. He did not respond to pharmacotherapy, and hepatic failure and renal insufficiency developed. Finally, a left ventricular assist device (LVAD) had to be implanted. Renal function normalized immediately as the cardiac output increased. Although such a case is exceptional, prerenal dysfunction is commonly observed even in patients with mild heart failure [2]. In case of heart failure and the resulting cardiac output decrease, the effective volume of circulating blood is reduced, baroreceptors are stimulated and excite the sympathetic nervous activity. Besides, renin secretion from the juxtaglomerular apparatus is enhanced due to the fall of renal perfusion pressure. Both norepinephrine and angiotensin II increase Na reabsorption from the proximal tubules and constrict glomerular mesangial cells decreasing the filtration area. Furthermore, angiotensin II increases the filtration fraction due to the constrictive action of efferent arterioles; as a result, plasma colloid oncotic pressure in the efferent arteriole rises leading to an increase of Na reabsorption from renal tubules. Besides, redistribution of blood flow from the cortical nephron to the juxtamedullary nephron, whose Na reabsorption ability is enhanced, results in fluid retention. Angiotensin II-induced secretion of aldosterone from adrenal zona glomerulosa cells promotes Na reabsorption from cortical collecting tubules. Furthermore, the decrease of effective intraarterial blood volume stimulates secretion of vasopressin (AVP) and causes water retention. Thus, the renin-angiotensin system (RAS), sympathetic nervous system and AVP are mutually activated in a vicious circle (Fig. (**3**)).

The natriuretic peptide family improves the balance of these neurohumoral factors. Atrial natriuretic peptide (ANP) and brain natriuretic peptide (BNP) inhibit aggravation of heart failure through vasodilatory and natriuretic actions. Also, natriuretic peptides antagonize the vasoconstrictor action and fluid retention action of RAS, sympathetic nervous system and AVP. The secretion of both ANP and BNP increases remarkably in heart failure, but the action is not potent enough to completely reduce edema or improve cardiac functions.

In heart failure, renal blood flow decreases due to the decline of cardiac output, but the GFR remains relatively constant. This is because intraglomerular pressure is elevated by the activation of RAS and the sympathetic nervous system. Because natriuretic peptides constrict efferent arterioles, while they dilate the afferent arterioles, GFR is relatively constant at this point. Moreover, the elevation of venous pressure in heart failure results in an increase of vascular resistance in the glomerular efferent side.

In the compensated state of heart failure, a constant GFR and fluid volume increase result in an increase of cardiac output, whereas in the decompensated state they lead to a reduced cardiac output and an increase of congestion. If renal hypoperfusion persists, it finally leads to ischemic injury of renal tissue. On the other hand, elevation of the intraglomerular pressure for a long time may induce an increase of proteinuria and glomerulosclerosis in patients who have already suffered glomerular damage. Furthermore, expression of proinflammatory cytokines such as TNF-α, IL-1ß or IL-6 increases in heart failure, and in fact, their levels are elevated in circulating blood. It is possible that cytokines contribute to the deterioration of the clinical condition of patients with heart failure although anticytokine therapies for heart failure have not proved successful so far. Renal vascular inflammation may occur likewise in heart failure. On the other hand, the blood level of asymmetric dimethylarginine (ADMA), a potent inhibitor of NO synthase, is increased in heart failure [14]. In addition to the above mentioned cytokines, NO synthase inhibition by ADMA causes endothelial damage in the renal vasculature.

## 4. POSSIBLE RENAL DAMAGE BY AGENTS USED TO TREAT AND DIAGNOSE CVD

### a) Contrast Media-Induced Nephropathy

Contrast media is the most important agent involved in the progression of renal damage in patients with CVD. Hypotension, congestive heart failure, less than 60 mL/min/ 1.73m^2^ of GFR, 75 years old or older, anemia, and diabetes mellitus are risk factors for contrast media-induced nephropathy. Most patients with CVD present some of these risk factors. Hydration with physiological saline most effectively prevents contrast media-induced nephropathy at present [15]. In addition, it has been reported that this type of nephropathy can be prevented by various methods including hemodialysis, dopamine, ANP, etc, but there is no consensus in this regard. The utility of elimination of reactive oxygen species by N-acetylcysteine and the dose-dependent efficacy of this amino acid were recently reported [16]. Hydration combined with the administration of N-acetylcysteine is recommended because it does not cause severe side effects. Furthermore, concomitant administration of bicarbonate with N-acetylcysteine also prevents increases in serum creatinine [17].

### b) Cholesterol Crystal Emboli

Fig. (**4**) shows a photograph of the biopsed skin from a 68-year-old man [18]. The patient showed progressive renal dysfunction and eosinophilia, and ultrasonography revealed an important degree of aortic atheromatosis. Cholesterol crystal emboli were observed in the arterioles. Cholesterol emboli in this case occurred spontaneously. However, it is highly likely that cholesterol crystal emboli are easily induced by cardiac catheterization in such cases with systemic atherosclerosis. Cholesterol emboli may also be involved in renal damage considered to be induced by contrast media.

## TREATMENT FOR PATIENTS WITH CARDIORENAL SYNDROME

### a) Renal Failure in Large-Scale Clinical Trials for CVD

It was shown in the subgroup analyses of major large-scale clinical studies such as ALLHAT (hypertension) [19], VALIANT (myocardial infarction) [20] and CHARM (heart failure) studies [21] that cardiovascular events increased as renal function decreased. In the subgroup analysis of the ALLHAT study [19] the endpoints were analyzed after subjects were distributed into 3 groups with an estimated GFR of less than 60 mL/min/1.73m^2^, 60-90 mL/min/1.73m^2^ and more than 90 mL/min/1.73m^2^. The incidence of renal events (end-stage renal failure [ESRD] or decrease in estimated GFR by more than 50%) was higher as the estimated GFR decreased, but there was no difference among three kinds of medication groups; namely, diuretics, Ca channel blockers and ACE inhibitors. However, cardiovascular events occurred much more frequently than renal events. There was no difference in the effect of the medication on the onset of CVD in patients with any degree of renal dysfunction. Therefore, it is possible that the difference in protective effects among the medication groups is small for progress of renal disease, at least in patients with hypertension. In this situation the blood-pressure level attained rather than the class of antihypertensive agent is more important to prevent cardiovascular events, as previously pointed out.

### b) Inhibitors of the Renin-Angiotensin-Aldosterone System (RAAS)

The administration of drugs that protect both the heart and kidney is reasonably desirable for patients with cardiorenal syndrome. Among them, the utility of RAAS inhibitors is easy to understand based on the role of RAAS in the pathogenesis of cardiac and renal damage. Aldosterone has recently been recognized as a substance that causes wide-range organ damage to an extent similar to that induced by angiotensin II. Actually, it was clearly proved by the RALES study [22] that spironolactone, an aldosterone receptor blocker, improved the prognosis of patients with heart failure. Moreover, spironolactone reduced proteinurina in some patients with the glomerular disease [23]. It has been recently shown that aldosterone increases the release of reactive oxygen species [24]. Its inhibition may explain at least in part the organ protection of the aldosterone antagonist. In addition to the blood-pressure lowering effect of ACE inhibitors and ARBs [25], the effect of RAAS inhibitors on glomerular microcirculation has been proposed as one of the mechanisms of renal protection. In other words, angiotensin II raises intraglomerular pressure because it constricts more potently efferent than afferent glomerular arterioles, and thereby it promotes glomerulosclerosis. It was also shown that aldosterone exerted direct vasoconstrictor activity in the glomeruli. The effect of aldosterone on efferent arterioles is more potent than on afferent ones. However, this action of aldosterone is nongenomic [26]. Further studies are required to confirm it.

### c) Principles for the Treatment of Cardiorenal Syndrome

Treatment should aim at attaining a strict control of the risk factors in patients with cardiorenal syndrome. Especially, an optimal blood pressure of 120/80 mmHg [27] should be aimed, although there is no clear evidence of its advantage. A positive correlation between baseline blood pressure and renal dysfunction exacerbation rate was found in 98,759 residents of Okinawa from 1983 to 2000, at the time of their medical check up. The incidence of ESRD was significantly elevated even in patients with a blood pressure slightly higher than normal (130-140/85-90 mmHg) [28]. In the subgroup analysis of the CAMELOT study population under treatment with various kinds of antihypertensive medications [29], the atheromatous plaque volume in the coronary arteries was larger in patients with a blood pressure of 140/90 mmHg or higher, did not change in those with 120-140/80-90 mmHg and decreased significantly in those with a blood pressure below 120/80 mmHg. According to these results, the target blood pressure in high-risk patients with both cardiac and renal damage should be the optimal one rather than just lower than 140/90 mmHg. A strict control of risk factors such as lipids, blood glucose or smoking is likewise necessary.

## CONCLUSION

It has been clarified that the presence of CKD tends to be associated with CVD, and vice versa. It is crucial to understand the basic clinical condition of both organs well, and it is necessary to pay attention to iatrogenic etiologies. The presence of renal damage must always be considered when treating patients with CVD, the same as risk factors, which should be strictly controlled.

## Figures and Tables

**Fig. (1):**
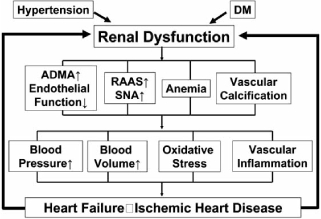
Proposed pathophysiological mechanisms for cardiorenal syndrome. DM: diabetes mellitus, RAAS: renin angiotensin alsosterone system, SNA: sympathetic nervous activity, ADMA: asymmetric dimethylarginine.

**Fig. (2):**
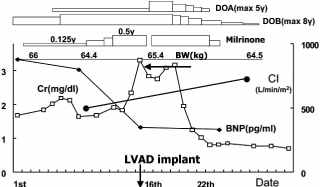
Time course changes in cardiac and renal functions in patients with dilated cardiomyopathy after implantation of left ventricular assist device (LVAD). DOA: dopamine, DOB: dobutamine, CI: cardiac index, Cr: creatinine, BNP: B-type natriuretic peptide.

**Fig. (3). Compensatory mechanisms for renal dysfunction and volume retention in heart failure.**
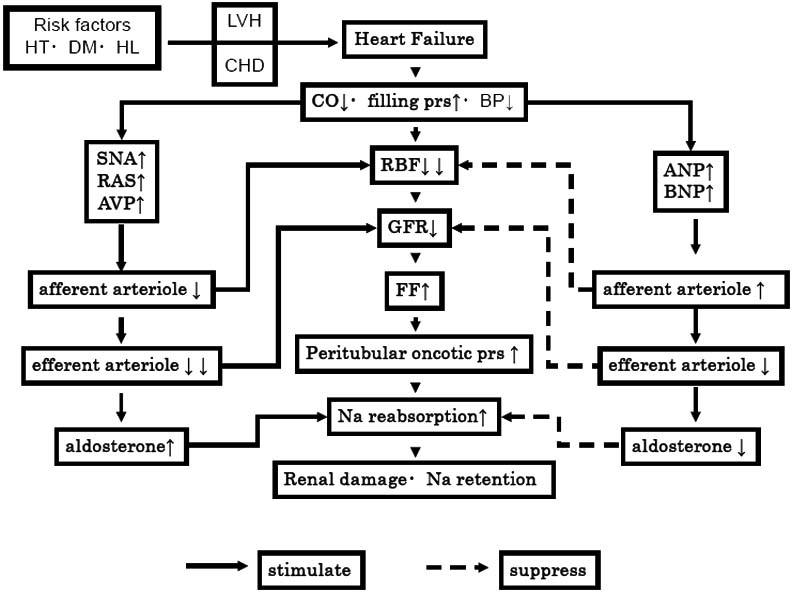
HT: hypertension, DM: diabetes mellitus, HL: hyperlipidemia, LVH: left ventricular hypertrophy, CHD: coronary heart disease, CO: cardiac output, prs: pressure, BP: blood pressure, SNA: sympathetic nervous activity, RAS: renin angiotensin system, AVP: arginine vasopressin, RBF: renal blood flow, GFR: glomerular filtration rate, FF: filtration fraction, ↓: decrease or constriction, ↑: increase or dilatation

**Fig. (4):**
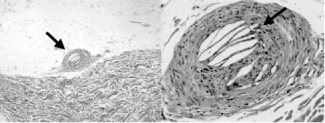
Photograph of the biopsed skin from a patient with cholesterol emboli. Arrow heads indicate cholesterol crystals. Taken from ref. [18]; Matsumura T, Hirata Y, *et al.* Am J Med Sci 2006; 331: 280-3.
